# Molecular Cytogenetic Characterization of New Wheat—*Dasypyrum breviaristatum* Introgression Lines for Improving Grain Quality of Wheat

**DOI:** 10.3389/fpls.2018.00365

**Published:** 2018-03-19

**Authors:** Hongjin Wang, Hongjun Zhang, Bin Li, Zhihui Yu, Guangrong Li, Jie Zhang, Zujun Yang

**Affiliations:** ^1^School of Life Science and Technology, University of Electronic Science and Technology of China, Chengdu, China; ^2^Center of Informational Biology, University of Electronic Science and Technology of China, Chengdu, China

**Keywords:** *Dasypyrum*, glutenin, FISH, grain quality, wheat

## Abstract

As an important relative of wheat (*Triticum aestivum* L), *Dasypyrum breviaristatum* contains novel high molecular weight glutenin subunits (HMW-GSs) encoded by *Glu-1Vb* genes. We identified new wheat—*D. breviaristatum* chromosome introgression lines including chromosomes 1V^b^ and 1V^b^L.5V^b^L by fluorescence *in situ* hybridization (FISH) combined with molecular markers. We found that chromosome changes occurred in the wheat—*D. breviaristatum* introgression lines and particularly induced the deletion of 5BS terminal repeats and formation of a new type of 5B-7B reciprocal translocation. The results imply that the *D. breviaristatum* chromosome 1V^b^ may contain genes which induce chromosomal recombination in wheat background. Ten putative high molecular weight glutenin subunit (HMW-GS) genes from *D. breviaristatum* and wheat—*D. breviaristatum* introgression lines were isolated. The lengths of the HMW-GS genes in *Dasypyrum* were significantly shorter than typical HMW-GS of common wheat. A new y-type HMW-GS gene, named *Glu-Vb1y*, was characterized in wheat—*D. breviaristatum* 1V^b^ introgression lines. The new wheat—*D. breviaristatum* germplasm displayed reduced plant height, increased tillers and superior grain protein and gluten contents, improved gluten performance index. The results showed considerable potential for utilization of *D. breviaristatum* chromosome 1V^b^ segments in future wheat improvement.

## Introduction

The high-molecular weight glutenin subunits (HMW-GS) are well-conserved endosperm proteins in the grain of wheat and related genus or species (Lawrence and Shepherd, 1981; Shewry et al., 2003). Much attentions have been paid over several decades to demonstrate that the HMW-GS can largely influence grain end-use quality (Shewry et al., 1992). Although the HMW-GS encoded by the loci *Glu-1* on long arm of wheat homoeologous chromosome group 1 only represent about 10% of grain protein (Payne et al., 1980), the variation of some allelic compositions of *Glu-1* loci were firmly comfirmed to be associated with bread-making quality (Anderson and Greene, 1989; Halford et al., 1992). A number of *Glu-1* genes from Triticeae species have been sequenced (Shewry et al., 2003; Liang et al., 2015), and the efforts have also been made to transfer *Glu-1* alleles from related species by wide hybridization and chromosomal engineering approaches (Mackie et al., 1996; Cao et al., 2007; Garg et al., 2009a; Li et al., 2013). Novel *Glu-1* loci from wheat relatives were also demonstrated to have positive effects on wheat improvement of end-use quality (Garg et al., 2009b). Meanwhile, the well-studied HMW-GS genes provide a useful model for the study of genetic variation during the process of wheat-alien hybridization and the development of introgression lines (Liu et al., 2008; Yuan et al., 2014).

*Dasypyrum* species possess agronomically important genes controlling traits including disease resistance, high protein contents and drought adaptation, which represent valuable resources for wheat improvement over the world (Zhang et al., 2005; De Pace et al., 2011). The annual species *Dasypyrum villosum* carries effective resistance genes to several fungal pathogens of wheat and has genes which may have a positive contribution to quality improvement when transferred into a wheat background (Chen et al., 1995; Patrizia et al., 2010; Qi et al., 2011; Zhao et al., 2010, 2015). It is thus essential to localize the novel seed proteins from *D. villosum* and to characterize the HMW-GS genes from wheat—*D. villosum* derivatives (Zhong and Qualset, 1993; Vaccino et al., 2010; Zhang et al., 2014; Yang et al., 2014). As an allotetraploid species of the genus *Dasypyrum*, *D. breviaristatum* (genome VVV^b^V^b^), has been previously studied with the aim of transferring useful genes into wheat (Yang et al., 2005; Baum et al., 2014) and such research has included the identification and localization of resistance genes on specific *D. breviaristatum* chromosomes (Li et al., 2014, 2016a). Meanwhile, the characterization of wheat—*D. breviaristatum* chromosomes derivatives has also revealed the genomic divergence between *D. breviaristatum* and *D. villosum*.

The aim of this study is to characterize new wheat—*D. breviaristatum* derivative lines carrying novel HMW-GS by fluorescence *in situ* hybridization (FISH) and molecular marker analysis, and to isolate the *Dasypyrum* specific HMW-GS genes with related to *Glu-1* evolution in Triticeae species and wheat quality improvement.

## Materials and Methods

### Plant Material

*Dasypyrum breviaristatum* accession PI 546317 was provided by the National Small Grains Collection at Aberdeen, ID, United States. The wheat—*D. breviaristatum* partial amphiploid TDH-2 (2n = 42, genome AABBV^b^V^b^) has been previously described by Yang et al. (2005). Lines D2176, D2186 and D2533 were obtained from a BC_1_F_6_ generation of the wheat variety Mianyang 11′ (MY11) crosses to wheat line ML19 and TDH-2 hybrids.

### Fluorescence *in Situ* Hybridization (FISH)

Chromosome preparation of mitotic metaphase from seedling root tips were followed the procedure of Han et al. (2006). In order to identify the wheat and *Dasypyrum* chromosomes, the sequences of synthesized oligo-nucleotide probes Oligo-pSc119.2, Oligo-pTa535 and Oligo- (GAA)_7_ referred the description of Tang et al. (2014) and Li et al. (2016a). The non-denaturing FISH (ND-FISH) with oligo probes was performed using the techniques described by Fu et al. (2015). The sequential FISH was conducted with long terminal repeat (LTR) pDb12H sequence (Yang et al., 2006; Liu et al., 2009) which was labeled by Alexa Fluor 488-5dUTP (Invitrogen) according to Han et al. (2006). The detection of FISH signals used an Olympus BX-51 Fluorescence microscope. The photography of FISH images was acquired with a DP-70 CCD camera.

### Glutenin Separation and PCR Cloning of HMW-GS Genes

The seed storage proteins including HMW-GS were extracted and examined by SDS-PAGE using the protocol described by Li et al. (2013). A pair of degenerate primers P1 (ATGGCTAAGCGGC/TTA/GGTCCTCTTTG) and P2 (CTATCACTGGCTG/AGCCGACAATGCG) for amplifying conserved HMW-GS genes were designed according to Xia et al. (2003). The target PCR products were cloned to pGEM-T vector (Promega) and sequenced by an automatic DNA sequencer (TaKaRa Biotech, Japan). The sequence alignment and phylogenetic tree construction were carried out by MEGA 4.0 software (Tamura et al., 2007).

### Grain Quality Analysis

The plant harvest for agronomic traits observations were collected from two field replications at the Xindu Experimental Station, Chengdu, China during the 2014–2017 seasons. Each 10 plants were grown in 1 m rows with 30 cm spacing between adjacent rows. The seeds quality were evaluated for each entry and plot with three replications. The protein content, wet gluten content, Zeleny sedimentation value, test weight, water absorption and of whole grains were determined using the near-infrared spectroscopy DA7250 (Perten, Sweden), according to the approved methods at Northwest A&F University, China. For testing solvent retention capacity (SRC) values, grain samples were milled using Brabender Quadramat milling system. The SRC tests were performed as described by Bettge et al. (2002). For 0.2 g wheat meal SRC, the deionized water, lactic acid solution (5% w/w), sucrose (50% w/w) and Na_2_CO_3_ solution (5% w/w) were tested for SRC-WA, SRC-LA, SRC-SU, SRC-SC, respectively. The gluten performance index (GPI) was defined as lactic acid/(sodium carbonate + sucrose) SRC values (Kweon et al., 2011). Each grain samples were measured in triplicate. Results of statistic data were analyzed by using SPSS software (version 22.0, SPSS, Chicago, IL, United States).

## Results

### Identification of *D. breviaristatum*-Specific HMW-GSs

**FIGURE 1:**
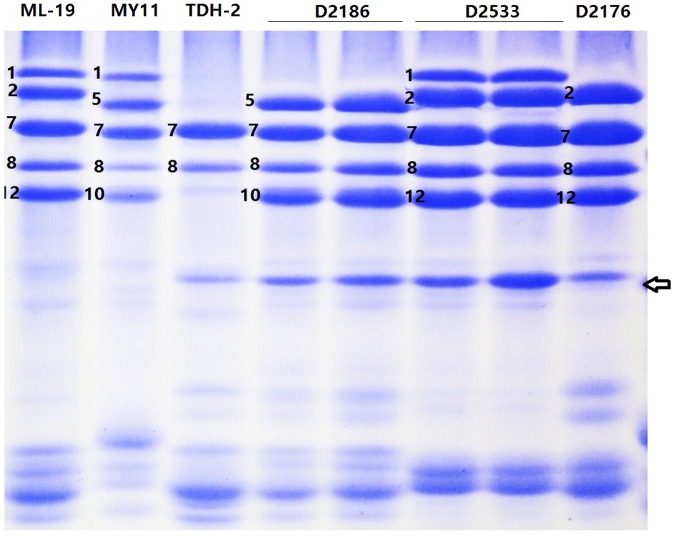
SDS-PAGE of seeds protein from wheat—*D. breviaristatum* lines and their parents. The arrow indicates the *D. breviaristatum* specific band. Electrophoretic profile indicated *D. breviaristatum* specific protein introduced to lines D2176, D2186 and D2533.

The SDS-PAGE analysis of seed storage protein extracts revealed that the wheat—*D. breviaristatum* partial amphiploid TDH-2 displayed wheat HMW-GS 7+8 of *Glu-B1* (**Figure 1**). TDH-2 contained the entire V^b^ chromosome and did not possess any chromosomes of the D-genome. The TDH-2 partial amphiploid had a strong glutenin subunit band with a fast electrophoretic mobility between the HMW-GS and LMW-GS regions (**Figure 1**, indicated by arrow). This subunit was also present in wheat—*D. breviaristatum* introgressions D2176, D2186 and D2533, but absent in other addition and control wheat parents ML-19 and MY11 (**Figure 1**). The unusual subunit was presumed to have originated from *D. breviaristatum* and expressed in the wheat—*D. breviaristatum* introgressions. Thus, we designated the additional bands in **Figure 1** as a *D. breviaristatum* specific *Glu-1* glutenin subunit.

### FISH of Wheat—*D. breviaristatum* Derivatives and Its Parents

**FIGURE 2:**
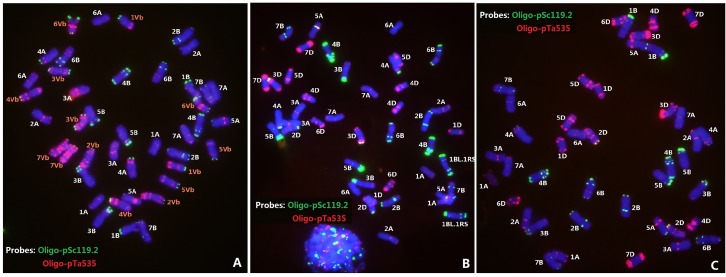
Fluorescence *in situ* hybridization (FISH) of wheat—*D. breviaristatum* partial amphiploid TDH-2 **(A)**, and wheat line ML-19 **(B)** and MY11 **(C)**. FISH with probes of Oligo-pSc119.2 (green) + Oligo-pTa535 (red) enables to precisely distinguish individual wheat and *D. breviaristatum* chromosomes.

**FIGURE 3:**
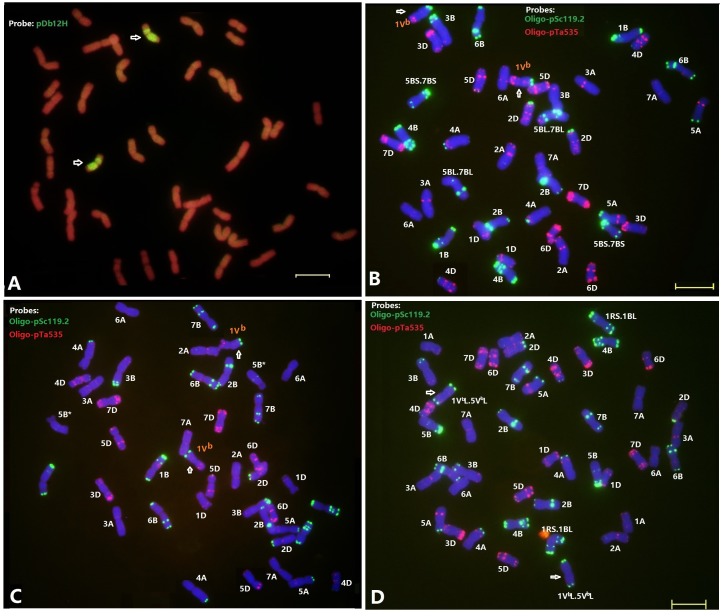
Fluorescence in situ hybridization of the wheat—*D. breviaristatum* introgression lines D2176 **(A,B)**, D2186 **(C)**, and D2533 **(D)**. The probe for sequential FISH **(A)** was pDb12H (green), while probes for FISH **(B–D)** were Oligo-pSc119.2 (green) and Oligo-pTa535 (red). Arrows showed the *D. breviaristatum* chromosomes. Stars represent the modified FISH signals for chromosome 5B. Bars indicate 10 μm. FISH analysis indicates that D2176 (2n = 42) and D2186 (2n = 42) were *D. breviaristatum* chromosome 1V^b^ substitute wheat chromosome 1A, D2533 (2n = 44) was a addition with *D. breviaristatum* translocated chromosomes.

Since the above identified lines carried *D. breviaristatum* specific glutenin bands, sequential multi-color ND-FISH by probes Oligo-pSc119.2, Oligo-pTa535 and Oligo-(GAA)_7_ was used to characterize the chromosome constitution of wheat—*D. breviaristatum* lines D2176, D2186 and D2533, in comparison with their parents TDH-2, ML-19 and MY11 (**Figures 2**, **3**). The 28 wheat chromosomes and seven pairs of *D. breviaristatum* chromosomes can be identified using probes Oligo-pSc119.2 and Oligo-pTa535 in TDH-2 (**Figure 2A**). The wheat parents ML-19 and MY11 were also characterized by multi-color ND-FISH using probes Oligo-pSc119.2 and Oligo-pTa535. The results showed that lines ML-19 carried a pair of 1RS.1BL translocation chromosomes (**Figure 2B**), while MY11 did not (**Figure 2C**).

The FISH using the *Dasypyrum* specific LTR probe (Yang et al., 2006) indicated the presence of a pair of *D. breviaristatum* chromosomes in the wheat—*D. breviaristatum* lines D2176 (**Figure 3A**). Sequential FISH revealed that *D. breviaristatum* chromosomes possessed specific and distinctive bands using probes Oligo-pSc119.2 and Oligo-pTa535. The chromosome number of D2176 was 2n = 42, in which a pair of chromosomes 1A was absent, and a pair of *D. breviaristatum* chromosomes had been added into the wheat background. The FISH revealed that 2176 contained a pair of alien chromosomes, which displayed a faint Oligo-pSc119.2 hybridization signals at the telomeric region of the long arms, and strong hybridization signals of Oligo-pTa535 in its short arm (**Figure 3B**). The FISH hybridization pattern of the chromosomes was identical to *D. breviaristatum* chromosomes 1V^b^ (**Figure 2**). Therefore, we conclude that the line D2176 was a wheat—*D. breviaristatum* chromosome 1V^b^ substitution line. Comparing the FISH patterns of D2176 parents MY11 and TDH-2 (**Figure 2**), we found that the D2176 line contained a wheat chromosomal 5B-7B non-Roberstanian translocation. Similarly, FISH revealed that line D2186 with 2n = 42 contained a pair of 1V^b^ chromosome substituted for chromosome 1A of wheat. The wheat chromosome 5B was shown to lack any strong Oligo-pSc119.2 signals at the telomeric regions of short arms (**Figure 3C**). Since there are no known such translocated chromosomes in the partial amphiploid TDH-2 and the wheat parents, the changed karyotypes of D2176 and D2186 could have arisen from induced chromosome breakage associated with the introgression of *D. breviaristatum* chromosome in the wheat background. The FISH hybridization pattern of the chromosomes in D2533 with 2n = 44 chromosomes indicated that it contained a pair of 1RS.1BL translocation chromosomes which had been inherited from the ML-19 parent, and also a pair of *D. breviaristatum* chromosomes (**Figure 3D**). After comparing the FISH patterns of these *D. breviaristatum* chromosomes with TDH-2 karyotype, we found that the unknown alien chromosomes consisted of two long arm of translocated but unidentified chromosomes. Since the FISH patterns of 1V^b^L, 2V^b^L, 3V^b^L, and 5V^b^L (Li et al., 2016a) are quite similar, molecular markers need to determine the linkage group(s) of the *D. breviaristatum* chromosomes in D2533.

#### PCR Analysis of the Wheat—*D. breviaristatum* Introgression Lines

**FIGURE 4:**
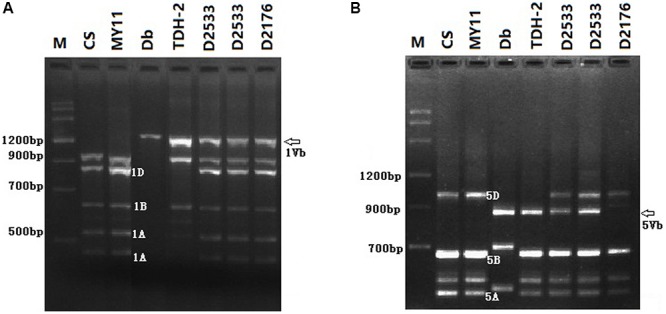
PCR analysis of markers TNAC 1021/*Taq*I **(A)** and TNAC1540/*Hae*III **(B)** to the test lines. CS- wheat “Chinese Spring”; MY11- wheat parent; Db- *D. breviaristatum*; TDH-2- wheat—*D. breviaristatum* partial amphiploid; D2533- 1V^b^L.5V^b^L translocation; D2176- 1V^b^ substitution. The arrows indicate the *D. breviaristatum* specific bands.

To identify the *D. breviaristatum* chromatin introduced in D2176, D2186, and D2533, PLUG markers were screened using PCR and the results compared with those of control wheat lines. A total of 18 and 32 markers previously located onto the short and long arms of wheat homoeologous group 1, respectively, were used to amplify DNA from the three introgression lines. We found that *D. breviaristatum* specific bands from both the short and long arms of group 1 could be visualized in D2176 (**Figure 4A**), however, only the long arm of group 1 specific bands could be amplified in D2533. The results showed that the D2176 and D2186 were wheat—*D. breviaristatum* 1V^b^ introgression lines, while only the 1V^b^L arm had been introduced to D2533. A total of 10 markers were located on the long arm of *D. breviaristatum* chromosome 5V^b^ and were mapped to the line D2533 (**Figure 4B**), which is similar to our previous study with chromosome 5V^b^L of *D. breviaristatum* (Zhang et al., 2015). The results showed that the 5V^b^L markers were present in D2533. In combination with the FISH analysis of D2533 (**Figure 3C**), we tentatively concluded that the *D. breviaristatum* chromosome in D2533 was a 1V^b^L.5V^b^L rearranged chromosome.

### Isolation of *D. breviaristatum Glu-1*

**FIGURE 5:**
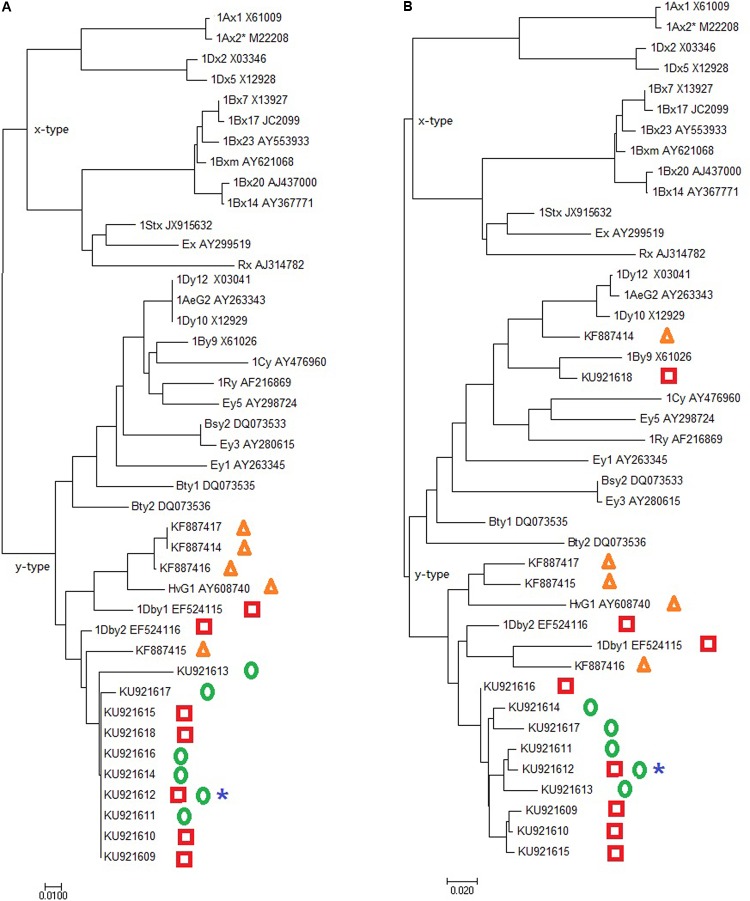
Phylogenetic tree of *Glu-1* genes based on the N-terminal region **(A)** and C-terminal **(B)**. The sequences from *D. villosum, D. breviaristatum,* TDH-2 and D2176 HMW-GS genes are marked as triangles, squares, circles and stars, respectively.

The AS-PCR primers P1 and P2 corresponding to the *Glu-1* amplicon were used to amplify genomic DNA samples of *D. breviaristatum* and TDH-2. The amplicon was subjected to cloning and a total of 20 clones from each template were sequenced. A total of 13 different sequences were found to be highly similar to the *Glu-1* consensus by Blast to NCBI database. Three sequences contained two in-frame stop codons and represented pseudogenes, and other ten sequences with intact ORFs encoding from 307 to 579 residue polypeptides, were deposited in Genbank under the accession numbers KU921609 to KU921618, respectively. Based on the prediction of the amino acid sequences, the *Dasypyrum*-specific subunit protein contained a signal peptide, an N-terminal domain, a central repetitive domain, and a C-terminal domain. The predicted cysteine number from five to nine was observed in the *D. breviaristatum* specific HMW-GS like sequences. In addition to the four *D. villosum* sequences (KF887414-KF887417) reported by Yang et al. (2014) and our previously cloned sequences EF524115 and EF524116, a total 16 *Dasypyrum* HMW-GS sequences were used to perform the phylogenetic analysis. As shown in **Figure 5**, the phylogenetic tree revealed that all *D. breviaristatum* and *D.* *villosum* HMW-GS sequences belonged to the y-type. The relative variations of the y-type of *Dasypyrum* HMW-GS sequences will provide the basis of interesting future studies test their effect on the quality improvement in wheat.

To determine whether the novel *D.* *breviaristatum* genes were transferred to the wheat—*D.* *breviaristatum* introgression lines, AS-PCR was also performed to amplify the DNA of D2176. The PCR products of 1.6-kb bands of D2176 were obtained and sent for sequencing. Besides the wheat *Glu-1* fragments, we found that D2176 give rise to a sequence identical to *D.* *breviaristatum* specific to the intact 1,536-bp ORF (with Genbank number KU921612), which was identified in both the tetraploid *D.* *breviaristatum* and the wheat—*D.* *breviaristatum* partial amphiploid TDH-2. As shown in **Supplementary Figure S1**, the first 21 residues function as a signal peptide. Five cysteine residues were found in the 88 amino acids of N-terminal domain. The *Glu-1* gene included the repetitive domains with two decapeptides, 13 nonapeptides, 32 hexapeptides, and two tripeptides. Both the number and positions of the cysteine residues in the N-terminal domain highly resembled those of typical y-type HMW subunits (**Supplementary Figure S1**). The *D.* *breviaristatum* *Glu-1*, named *Glu-Vb1y*, is consistent with the molecular weight indicated by SDS-PAGE results (**Figure 1**), suggesting that the target *D. breviaristatum* specific HMW-GS subunit was introduced to wheat.

### Agronomic Trait Studies

**FIGURE 6:**
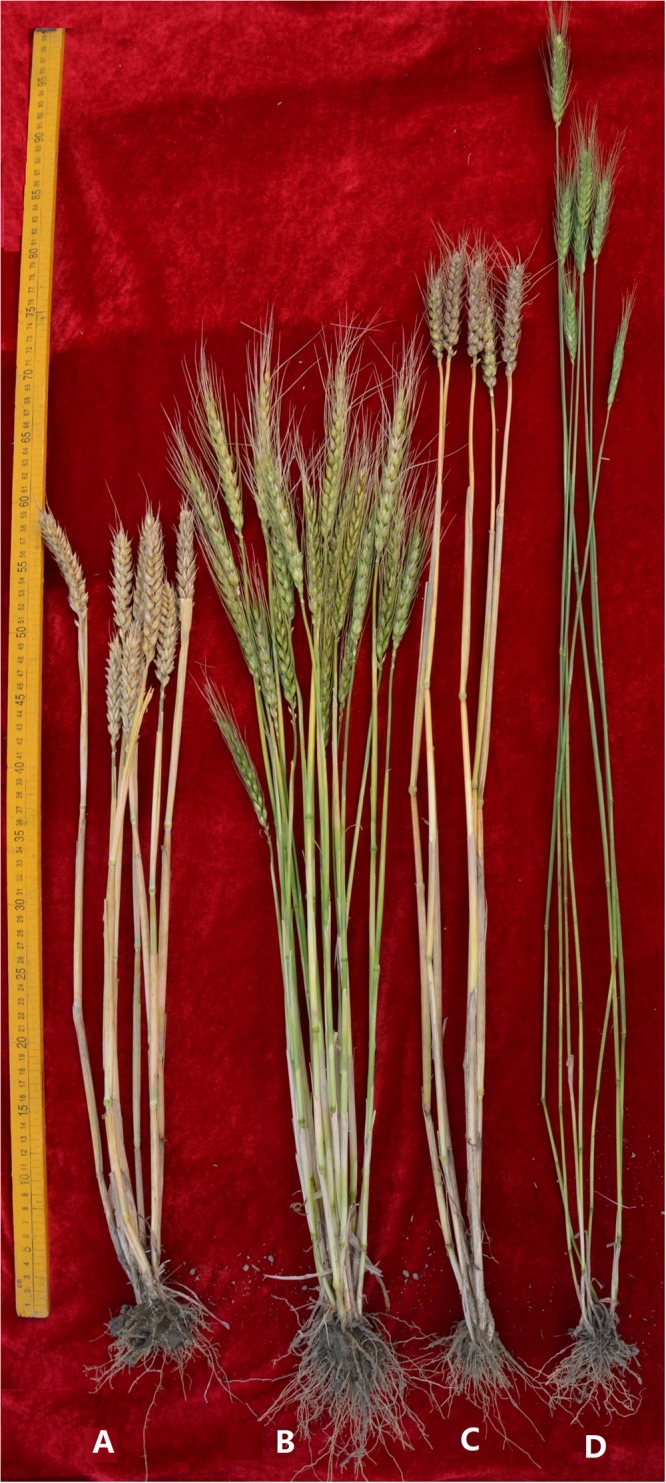
The adult plant morphology of the lines D2533 **(A)**, D2176 **(B)**, MY11 **(C)** and TDH-2 **(D)**.

Agronomic traits were measured on plants of the introgression lines D2176, D2533 and their parents grown in the field during 2015 and 2016 seasons. As shown in **Figure 6** and **Table 1**, relative to the plant height of TDH-2 (102 cm), MY11 (80 cm) and ML-19 (95 cm), D2176 and D2533 lines had reduced plant height of 60 and 72 cm, respectively, suggesting that chromosome 1V^b^L may carry a dwarfing gene(s) expressed in the wheat background. The tiller number per plant was significantly increased in D2176 (averaged 14) than D2533 (averaged 7) implying that the gene(s) on chromosome 1V^b^S may enhance the tiller development although the partial amphiploid TDH-2 showed only a few tillers (**Figure 6**). The line D2176 displayed low grain weight which may be caused by the late maturity inherited from its wheat—*D. breviaristatum* partial amphiploid TDH-2.

**Table 1:** Agronomic and grain quality traits of wheat—*Dasypyrum breviaristatum* lines and the wheat parents.

Lines	Plant height (cm)	Tillers per plant	Spike length (cm)	Spikelet per spike	Thousand kernel weight (g)
MY11	80.2 ± 4.30^a^	3.5 ± 0.8^a^	9.8 ± 0.9^a^	19.2 ± 1.2^a^	35.90 ± 2.1^a^
ML-19	95.3 ± 2.70^b^	4.0 ± 1.7^a^	9.5 ± 1.4^a^	19.6 ± 1.5^a^	39.65 ± 1.3^b^
D2176	60.7 ± 3.50^c^	14.5 ± 3.4^b^	10.5 ± 1.6^a^	20.4 ± 1.9^a^	32.67 ± 5.7^a^
D2186	62.5 ± 4.80^c^	10.0 ± 2.9^b^	11.2 ± 1.5^a^	20.3 ± 2.6^a^	33.78 ± 6.4^a^
D2533	72.1 ± 2.60^a^	7.0 ± 1.8^b^	10.9 ± 23^a^	22.2 ± 1.8^b^	41.40 ± 1.5^c^

*Means followed by the same letter within a column are not significantly different at *p* > 0.05*.

### Seeds Quality Test

**FIGURE 7:**
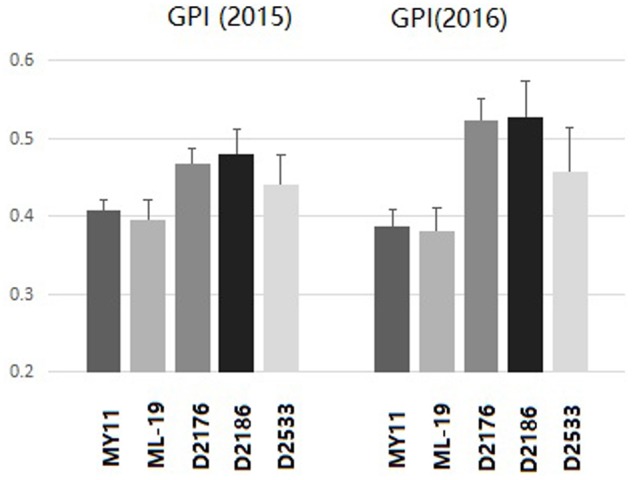
The gluten performance index (GPI) of flour for D2176, D2178, D2533 compared to wheat parents MY11 and ML-19 of 2015 and 2016. The GPI indicated the gluten quality improved in wheat—*D. breviaristatum* lines.

The grain protein content (GPC), wet gluten content (WGC), Zeleny sedimentation value (ZEL), water absorption (ABS) as well as the solvent retention capacity (SRC) were tested among D2176, D2186, and D2533 and their parents MY11 and ML-19. Both D2176 and D2186 showed a increased GPC, WGC, ZEL values, indicating higher dough strength compared to the wheat lines (**Table 2**). The SRC provides a measure of solvent compatibility for the three functional polymeric components of flour (gluten, damaged starch, and pentosans), reflecting wheat protein quality, starch quality and dough rheology characteristics on microscale (Bettge et al., 2002). As indicated in **Table 3**, D2176 and D2186 showed higher SRC-LA and lower SRC-SC, SRC-SU compared to those SRC values of control wheat during the both years of 2015 and 2016 (**Table 3**). The gluten performance index (GPI) were calculated based on the SRC values of the flour (Kweon et al., 2011). The results indicate a clear increase in GPI of lines D2176, D2186 and D2533 compared to those of wheat parents in both years (**Figure 7**). It suggested that the wheat—*D. breviaristatum* lines may display an overall flour performance for good finished-product quality.

**Table 2:** Quality parameters of wheat*—Dasypyrum breviaristatum* lines in comparison to the wheat parents.

Lines	Grain protein content (%)	Wet gluten content (%)	Zeleny value (mL)	Starch (%)	Water absorption	Test weight (g/L)
MY11	11.34 ± 0.30^a^	25.45 ± 1.22^a^	27.12 ± 1.66^a^	24.74 ± 1.87^a^	56.9 ± 3.76^a^	785 ± 3.4^a^
ML-19	11.87 ± 0.46^a^	27.10 ± 0.89^a^	28.33 ± 1.31^a^	35.48 ± 2.33^b^	57.5 ± 2.53^a^	773 ± 2.1^a^
D2176	16.32 ± 0.79^b^	36.61 ± 1.10^b^	41.92 ± 2.23^b^	40.24 ± 1.46^c^	60.9 ± 3.11^a^	718 ± 3.1^a^
D2186	15.57 ± 0.60^b^	33.96 ± 0.92^b^	38.59 ± 2.16^b^	43.26 ± 2.75^c^	60.2 ± 1.89^a^	749 ± 4.3^a^
D2533	14.86 ± 0.58^b^	29.60 ± 1.40^a^	32.34 ± 1.55^a^	34.86 ± 2.46^b^	63.1 ± 2.32^b^	784 ± 1.8^a^

*Means followed by the same letter within a column are not significantly different at *p* > 0.05*.

**Table 3:** Solvent retention capacity (SRC) comparison between wheat—*Dasypyrum breviaristatum* lines and their wheat parents by 2 years.

Lines	SRC-WA %	SRC-LA %	SRC-SC %	SRC-SU %	SRC-WA %	SRC-LA %	SRC-SC %	SRC-SU %
	(2015)	(2015)	(2015)	(2015)	(2016)	(2016)	(2016)	(2016)
MY11	78.7 ± 1.8^a^	94.3 ± 2.6^a^	101.5 ± 1.5^a^	130.3 ± 6.3^a^	91.6 ± 3.4^a^	104.5 ± 4.4^a^	121.3 ± 4.8^a^	148.2 ± 5.4^a^
ML-19	73.7 ± 2.7^a^	92.6 ± 2.0^a^	100.7 ± 2.1^a^	133.6 ± 7.1^a^	93.5 ± 3.6^a^	102.6 ± 3.5^a^	127.6 ± 5.2^a^	141.7 ± 4.8^a^
D2176	74.8 ± 2.2^a^	98.6 ± 2.0^b^	90.4 ± 1.6^b^	120.8 ± 4.5^b^	99.7 ± 2.9^b^	118.6 ± 3.2^b^	100.8 ± 3.7^b^	125.9 ± 6.5^b^
D2186	76.9 ± 3.9^a^	99.5 ± 3.1^b^	88.8 ± 2.4^b^	118.8 ± 6.4^b^	97.8 ± 4.3^b^	119.5 ± 4.4^b^	98.8 ± 5.3^b^	127.4 ± 4.9^b^
D2533	73.2 ± 2.0^a^	95.5 ± 3.2^a^	92.5 ± 2.9^b^	124.5 ± 6.2^b^	98.4 ± 2.5^b^	109.5 ± 6.3^a^	104.5 ± 5.9^b^	134.5 ± 5.7^a^

*SRC-WA, SRC-LA, SRC-SC, SRC-SU representing solvent retention capacity (SRC) by water, lactic acid, Na_2_CO_3_ solution, and sucrose solution, respectively. Means followed by the same letter within a column are not significantly different at *p* > 0.05*.

## Discussion

Fluorescence *in situ* hybridization (FISH) has been one of the most useful techniques for identifying chromosome constitution of wheat-alien derivatives (Jiang and Gill, 1993). The development of non-denaturing FISH (ND-FISH) based on synthetic labeled oligonucleotides, such as Oligo-pSc119.2 and Oligo-pTa535, enables a fast, low-cost and effective method to identify the *Secale*, *Dasypyrum,* and *Thinopyrum* chromosomes in a wheat background (Tang et al., 2014; Li et al., 2016a,b). Screening by FISH and GISH, a *D. breviaristatum* chromosome 2V^b^ substitution line D11-5 (Li et al., 2014), a 7V^b^ addition D2139 (Li et al., 2016a), and a 5V^b^L arm translocated onto wheat chromosome 5AS in D2146 (Zhang et al., 2015), were recently identified in a wheat background. In the present study, we screened by SDS-PAGE the HMW-GS seed proteins, and identified a *D. breviaristatum* specific *Glu-1* band introduced to wheat in lines D2176, D2186 and D2533 (**Figure 1**). The FISH and molecular markers confirmed that the *D. breviaristatum* derived HMW-GS was located on the 1V^b^L. The chromosome 1V^b^ of D2176 and D2186 contained a satellite region, which was detected by probe pTa71 in the short arm of 1V^b^ (data not shown); this result conforms to the structure of wheat chromosomes where NOR regions are commonly present in the short arm of homoeologous group chromosome 1. Since the agronomic traits observations revealed that the wheat—*D. breviaristatum* 1V^b^ lines displayed a higher tiller number than their wheat parents (**Figure 6**), it possibly implies that the short arm of *D. breviaristatum* chromosome 1V^b^ carries genes for enhancing the tiller development, which is similar to the reputed yield-enhancing effect of rye chromosome 1RS and hence possibly useful for yield improvement in a wheat background.

**FIGURE 8:**
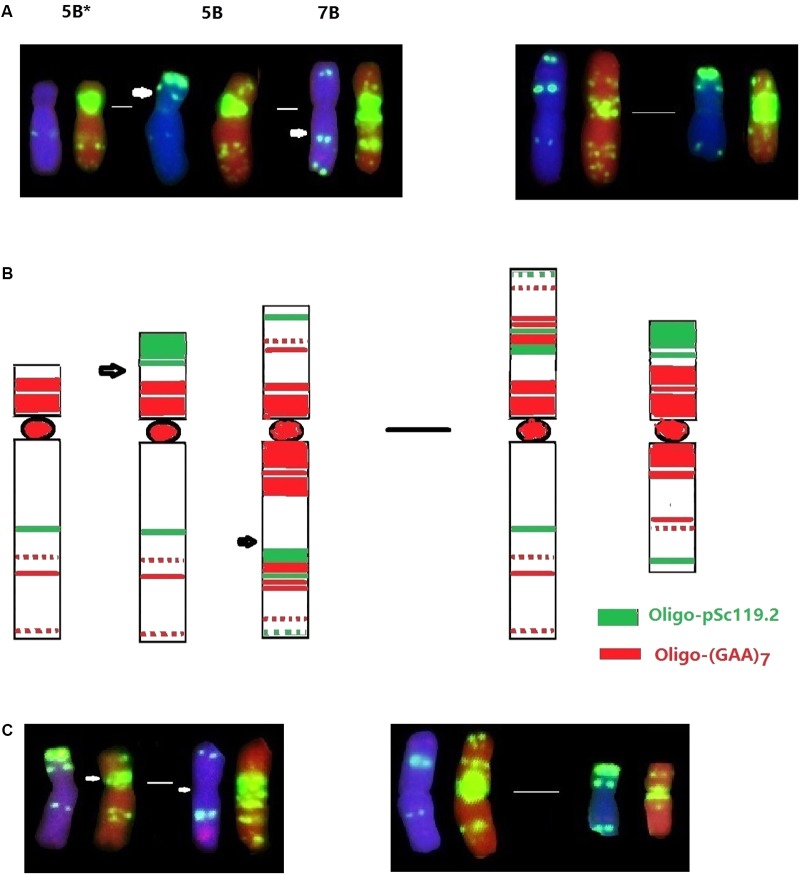
Comparison FISH karyotype of 5B deletion and 5B-7B translocations. The probes were Oligo-pSc119.2 (green) and Oligo-pTa535 (red). Arrows indicate the breakage points. The 5B deletion (5B^∗^) from D2176, the 5B-7B reciprocal translocation from D2186 indicated by FISH **(A)** and their karyotypes **(B)**. The 5B-7B reciprocal translocation cultivar Vilmorin-27 was showed **(C)**.

Interspecific hybridization combines divergent genomes into one nucleus, and is important for polyploidization and speciation by chromosome doubling of wide hybrids and introgression by subsequent backcrossing of the hybrids (Rey et al., 2015). Alterations of alien chromosomal structure and karyotypic variations of wheat chromosomes have previously been observed in the wheat-alien amphiploids, addition and substitution lines (Dou et al., 2006; Bento et al., 2010; Fu et al., 2013, 2015). Our recent studies also revealed the occurrence of apparent structural changes to chromosomes 1B, 2B, and 7A of the wheat—*D. breviaristatum* partial amphiploid TDH-2, and chromosomes 1D and 3D of the wheat—*D. breviaristatum* 7V^b^ addition lines by FISH (Li et al., 2016a). In the present study, the 5BS terminal Oligo-pSc119.2 signals were absent in D2186, and reciprocal translocations between the short and long arms of chromosomes 5B and 7B were observed in D2176 (**Figure 3**). The structure of the translocations were confirmed to be non-Roberstanian by FISH with probes of Oligo-pSc119.2 and Oligo-pTa535 (**Figure 2**), as well as Oligo-(GAA)7 (**Figure 8**). The breakpoints 5B-7B translocation in D2176 were located in the short arm of chromosome 5B distal to the sub-telomeric signals of Oligo-pSc119.2, and proximal the peri-centromeric signal Oligo-(GAA)7 in the long arm of chromosome 7B (**Figure 8**). The common wheat cultivar ‘Cappelle-Desprez’ with the 5B-7B reciprocal translocation was first reported by Riley et al. (1967) and was associated with durable rust resistance in European and Australian wheat lines (Law and Worland, 1997). The 5B-7B translocation is also present in a number of other wheat and durum wheats (Badaeva et al., 2007, 2014). The FISH of three Oligo probes also conducted to the French cultivar Vilmorin-27, with a 5B-7B translocation chromosome (**Figure 8C**). We indicated that the breakage point of 5B-7B in Vilmorin-27 was not identical to the 5B-7B chromosomes in D2176. We assumed that the new 5B.7B reciprocal translocation was induced by *D. breviaristatum* chromatin during the development of D2176, since no parent of D2176 contained such a translocation. Both chromosomes 5B and 7B contained rich heterochromatin in their centromeric and subtelomeric regions, the breakage and reunion easily happened in such repetitive sequence abundant regions (Molnár et al., 2011). Our results also supported that the 5B deletion in D2186 and 5B-7B translocation in D2176, which presumably induced by the introgression of *D. breviaristatum* chromatin. Recently, Gorafi et al. (2016) observed that insertions of miniature inverted-repeat transposable elements in the promoter region caused early flowering during the development of wheat—*Leymus* chromosome introgression lines. The results also support that the introgression of the chromosomes of wild species could promote genetic changes including both at the karyotypic level and sequence levels. The studies on the wheat-alien introgression lines provides opportunity to create the novel variations for future wheat breeding and the genetic studies to interpret the trait changes.

The wheat HMW glutenin locus and the orthologous loci are unique to Triticeae species suggests that these loci evolved relatively recently (Shewry et al., 1995; Gu et al., 2006). The alignments and phylogenetic trees of obtained *Glu-1* sequences from wheat and its related Triticeae species have revealed a remarkable conservation of these protein genes (Gu et al., 2004; Wang et al., 2007). Novel variant of HMW glutenin subunits genes of *Pseudoroegneria stipifolia* (Li et al., 2008; Zhang et al., 2016), *Thinopyrum elongatum* (Liu et al., 2008), *Th. intermedium* (Cao et al., 2014), and *Elymus glaucus* (Jiang et al., 2010) showed smaller size than those of conventional HMW-GS subunits due to the short length of their repetitive domain. It is reasonable to assume that the parallel evolution of gene copy number and the gene length amplification in the orthologous HMW-GS genes has occurred during the divergence of wheat and its relative species. Southern hybridization seems to be a feasible method for testing the copy number variation of *Glu-1* like loci in *Dasypyrum* genomes and their derivatives. The significant sequence changes occurred may due to the tetraploidization event of *Dasypyrum* speciation after the split of barley from Triticeae (Liu et al., 2010; Li et al., 2014). It is therefore noteworthy that the clear redundancy in both *D. breviaristatum* and *D. villosum* HMW-GS genes derived after a long period of cross-hybridization and polyploidization, may provide a clue to interpret the length of *Glu-1* sequences from wheat’s ancestors to those in modern wheat species.

The *Dasypyrum* genus contains only two species, which represent unique evolutionary entities in Triticeae tribe (De Pace et al., 2011). Based on the SDS-PAGE analysis and cytological identification of the wheat—*Dasypyrum* materials, Zhong and Qualset (1993) showed that a large variability exists for *Glu-V1* of *D. villosum*, and 14 alleles at *Glu-V1* were found in their observed *D. villosum* accessions. Genes at locus *Glu-V1* for HMW storage protein subunits are located on chromosome 1V, and prolamin genes are located on chromosomes 1V and 4V (Montebove et al., 1987; De Pace et al., 2001; Vaccino et al., 2010). The recently identified two wheat—*D. villosum* homozygous translocations confirmed that the HMW-GS gene of *D. villosum* was located on both 1VL and 1VS (Dong et al., 2013; Zhang et al., 2014; Zhao et al., 2015). In the present study, we also cloned ten sequences from *Dasypyrum* sequences and identified the different lengths of HMW-GS sequences in both *D. breviaristatum* and the wheat—*D. breviaristatum* partial amphiploid TDH-2. Only one sequence KU921012 named as *Glu-Vb1y* was found in the wheat—*D. breviaristatum* 1V^b^ line D2176. There also is the possibility that many of the *D. breviaristatum Glu-1* gene sequences may be located on other chromosomes other than 1V^b^. In particular, the *D. breviaristatum* derived *Glu-1* genes showed great variation in the length and number of cysteines, such as the sequence KU921611 with 1457bp containing nine cysteines. The *Glu-1Vby* gene of KU921611 contained seven cysteines which were transferred to D2176 in wheat background, and the additional number of cysteines of *Glu-1* may be potentially beneficial to bread-making quality. Yang et al. (2014) also found that the four HMW-GSs originated from *D. villosum* had positive effects on dough quality properties. In the present study, the effects of the new types of *D. breviaristatum* HMW-GS sequences to wheat quality were also confirmed that lines D2176 and D2186 relatively improved the flour quality of the wheat *D. breviaristatum* 1V^b^ lines by increasing the protein content, wet gluten contents, and gluten performance index from solvent retention capacity values. Previously, we identified a *Thinopyrum intermedium* ssp. *trichophorum* derived 1St#2L-specific *Glu-1St#2x* gene from a wheat—*Th. intermedium* 1St#2(1D) substitution line AS1677 which had a positive effect on wheat quality (Li et al., 2013). The *Glu-1St#2x* gene with ORF of 1,515 bp (Li et al., 2013), which was smaller than the present *D. breviaristatum* derived *Glu-1Vby* gene with ORF of 1,536 bp, although their protein located in the similar region by SDS-PAGE. It is thus to note that the small molecular weight HMW-GS genes from *Dasypyrum* and *Thinopyrum* chromosomes introgression can improve wheat end product quality.

## Author Contributions

ZuY and GL designed the experiments. HW, HZ, BL, and ZhY performed the experiments. GL and JZ analysis the data. ZuY and GL wrote the paper.

## Conflict of Interest Statement

The authors declare that the research was conducted in the absence of any commercial or financial relationships that could be construed as a potential conflict of interest.

## Supplementary Material

FIGURE S1 The nucleotide and amino acid sequence of *Glu-1Vby*. The cysteine residues are boxed and the unique repeat motifs of decapeptides, nonapeptides, hexapeptides, tripeptides in the central repetitive region were underlined by double, dash, single, triple lines, respectively.
