# Supplementary figures and images for: Molecular Cytogenetic Characterization of New Wheat—Dasypyrum breviaristatum Introgression Lines for Improving Grain Quality of Wheat
Source: Front Plant Sci. 2018 Mar 19;9:365. doi: 10.3389/fpls.2018.00365 (PMC5868130; doi:10.3389/fpls.2018.00365)

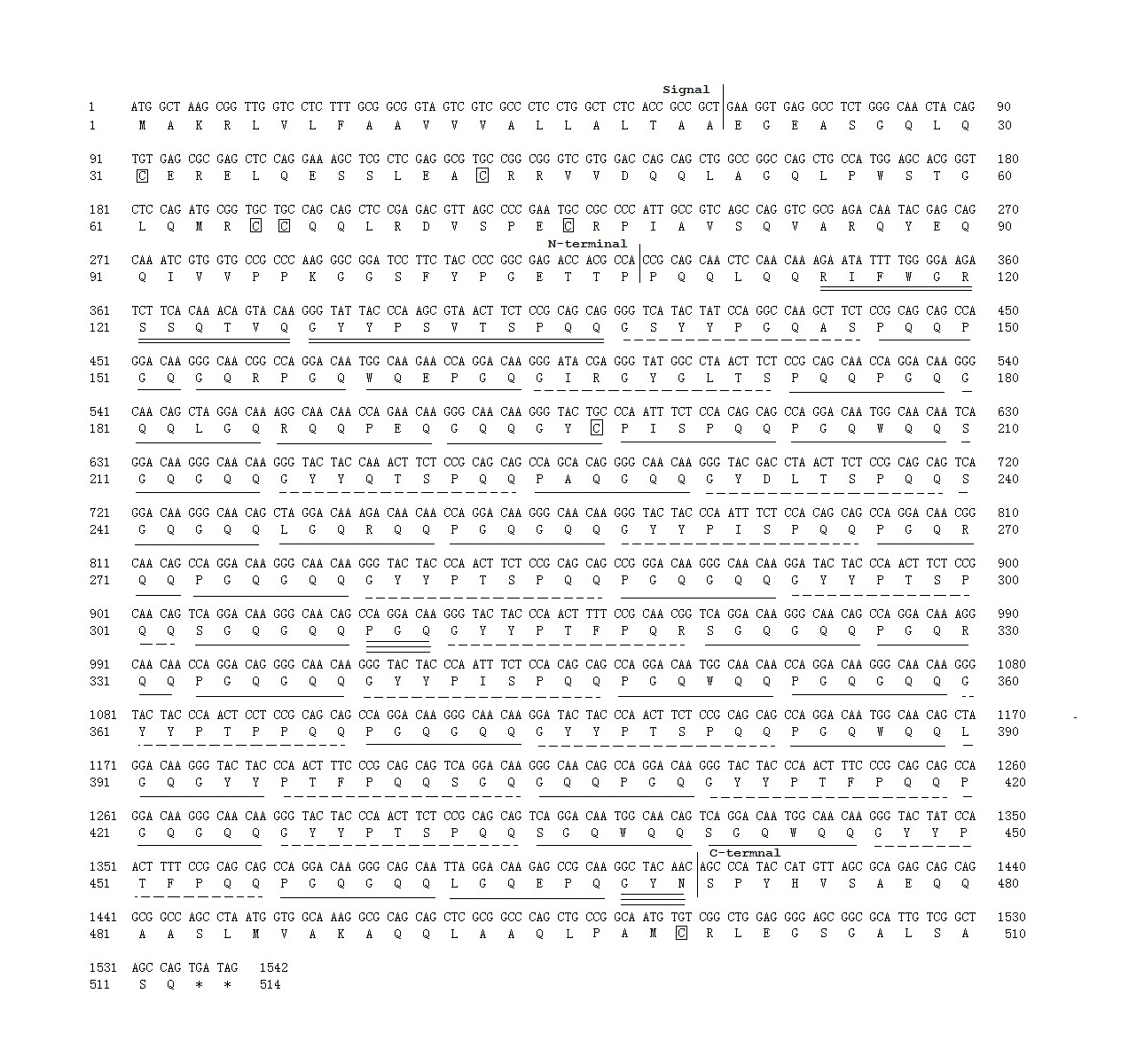

Supplement: FIGURE S1 — The nucleotide and amino acid sequence of Glu-1Vby. The cysteine residues are boxed and the unique repeat motifs of decapeptides, nonapeptides, hexapeptides, tripeptides in the central repetitive region were underlined by double, dash, single, triple lines, respectively. [file Image_1.JPEG]
